# Case Report: Severe asthma following earthquake-related inhalational exposure in a child

**DOI:** 10.3389/fped.2026.1893129

**Published:** 2026-08-24

**Authors:** Gaith Al-Zoubi, Mahmoud Nofal, Khaled El Wahab, Nizar Alkhlaifat

**Affiliations:** Department of Pediatric and Adolescent Medicine, Western Michigan University Homer Stryker M.D. School of Medicine, Kalamazoo, MI, United States

**Keywords:** dust inhalation injury, immigrant health, post-earthquake inhalation exposure, rubble entrapment, severe asthma

## Abstract

**Background:**

The 2023 Kahramanmaraş earthquakes in Türkiye generated massive quantities of respirable dust containing silica, heavy metals, and fungal spores. Children are uniquely vulnerable to disaster-related respiratory injury, yet detailed clinical characterization of pediatric survivors with extreme exposure remains limited.

**Case presentation:**

A 5-year-old girl presented to a U.S. community health center with progressive cough and dyspnea eight days after immigrating from Türkiye. She had been trapped under earthquake rubble for three days two years prior, during which her father died. Over five months, she required five hospitalizations for recurrent hypoxemic respiratory failure with oxygen saturations as low as 70%. CT chest demonstrated diffuse bronchial wall thickening, intraluminal debris, and air trapping. Laboratory evaluation revealed markedly elevated total IgE (1,664 kU/L) with a negative comprehensive respiratory allergen panel and peripheral eosinophilia (10%), while extensive infectious and alternative diagnostic workup was negative. Stepwise escalation to GINA Step 4–5 triple controller therapy (ICS-LABA, LAMA, LTRA) failed to prevent hospitalizations, with two admissions precipitated by medication access lapses. The patient was subsequently lost to follow-up.

**Conclusions:**

Prolonged earthquake rubble entrapment during early childhood may contribute to the development of severe asthma with non-allergen-specific IgE elevation and eosinophilia, compounded by virus-triggered exacerbations, immigration-related medication access barriers, and psychological trauma. Biologic therapy and systematic respiratory surveillance of pediatric earthquake survivors warrant consideration.

## Introduction

The February 6, 2023, Kahramanmaraş earthquake sequence in southeastern Türkiye was among the deadliest seismic events in modern history. Two earthquakes of magnitude 7.8 and 7.7 struck within nine hours, killing over 50,000 people, injuring more than 115,000, and displacing millions across eleven provinces (1–3). Beyond the immediate toll of structural collapse and traumatic injury, earthquakes generate massive quantities of respirable particulate matter from building destruction. Measurements taken during debris removal after the Kahramanmaraş earthquakes recorded average respirable dust concentrations of 30.84 mg/m^3^, with scanning electron microscopy identifying fibrous structures and elements associated with asbestos (4). Earthquake rubble dust is a complex mixture that may include crystalline silica, calcium compounds, heavy metals, and biological contaminants including fungal spores (5, 6).

The respiratory consequences of such exposures are well documented in analogous disaster settings. Following the September 11, 2001, World Trade Center collapse, dust cloud exposure was associated with new asthma diagnoses in children, with an adjusted odds ratio of 2.3, and children under 5 years of age were disproportionately affected, with post-disaster asthma prevalence exceeding national estimates (7, 8). Similarly, hospitalization rates for respiratory diseases increased significantly in the area surrounding the 2009 L'Aquila earthquake in Italy (9). More recently, a study of 528 children with allergic diseases following the Kahramanmaraş earthquakes found that demolition-related dust exposure (aOR 1.9, 95% CI 1.1–3.5) and prolonged residence in temporary housing (aOR 2.1, 95% CI 1.2–3.9) were independently associated with clinical deterioration, including worsening asthma control (10).

Children are uniquely vulnerable to disaster-related respiratory injury. Their higher minute ventilation relative to body weight, lower airway caliber, and developing immune systems predispose them to greater particulate deposition and airway inflammation. Children who survive building entrapment face an extreme exposure scenario: prolonged, high-concentration inhalation of crushed rubble dust in a confined space, compounded by physiological stress and potential aspiration. The long-term respiratory consequences of such exposures in pediatric populations remain incompletely characterized, with most existing data derived from population-level epidemiological studies rather than detailed clinical follow-up of individual survivors (5, 11).

An additional layer of complexity arises when disaster-affected children subsequently migrate to new countries. Immigrant and refugee children face well-documented barriers to chronic disease management, including insurance enrollment challenges, language discordance, limited health literacy, disrupted continuity of care, and lack of a medical home. These systemic barriers can transform a potentially manageable chronic condition into one characterized by recurrent acute decompensation (11, 12).

We report the case of a young girl who survived three days of entrapment under earthquake rubble and subsequently developed severe, difficult-to-control asthma requiring five hospitalizations over five months after immigration to the United States. This case illustrates the intersection of disaster-related environmental airway injury, the diagnostic challenge of markedly elevated IgE without allergen-specific sensitization in the context of massive dust exposure, and the compounding effects of social determinants on chronic disease management in an immigrant child.

## Case presentation

A 5-year-old girl presented to a community health center in the United States with progressive dry cough and difficulty breathing, eight days after immigrating from Türkiye. Two years prior, the family had been trapped under rubble for approximately three days during the 2023 Kahramanmaraş earthquakes; the patient's father died during this event. Following the earthquake, she developed intermittent coughing triggered by exercise, perfumes, and strong odors. She had been hospitalized in Türkiye for two months for acute respiratory failure but was not maintained on controller medications upon discharge. No records from this hospitalization were available for review. Pre-earthquake allergic, atopic, or respiratory history was not available, and family history of atopy or asthma could not be obtained from the surviving parent.

### Hospital course

Over the subsequent five months, the patient required five hospitalizations for recurrent hypoxemic respiratory failure. The clinical course, workup, and treatment escalation are summarized below and in Table 1.

**Table 1:** Summary of hospitalizations.

Admission	Presenting SpO_2_	Key diagnostic findings	Treatment changes
1st (Jan 2025)	Hypoxic on 10 L O_2_	RUL opacity; normal CRP/PCT; negative pathogen panel	Albuterol PRN only
2nd (Feb 2025)	70%–80%	CT: bronchial wall thickening, debris, air trapping; IgE 1,664 kU/L; rhinovirus+	Added ICS (fluticasone) + LTRA (montelukast)
3rd (Mar 2025)	77%–80%	Rhinovirus+; ABPA workup initiated; respiratory allergen panel obtained (Table 3)	Tiotropium planned; biologic therapy considered
4th (Apr 2025)	83%	RML atelectasis; medication nonadherence identified	Social work for medication access
5th (May 2025)	Hypoxic on NRB	Eosinophilia 10%; rhinovirus+; CF/PCD/A1AT negative	Escalated to ICS-LABA + LAMA + LTRA

Over the subsequent five months, the patient required five hospitalizations for recurrent hypoxemic respiratory failure. The clinical course, workup, and treatment escalation are summarized below, in Table 1, and in Table 2.

**Table 2:** Clinical timeline.

Time point	Event	Key details
February 2023	Kahramanmaraş earthquakes; rubble entrapment	Patient (age 3) trapped ∼3 days; father died
Feb–Apr 2023	Acute hospitalization in Türkiye	2-month admission for respiratory failure; no controller therapy at discharge; no records available
2023–2024	Interval period in Türkiye	Intermittent cough triggered by exercise, perfumes, strong odors; no controller medications
January 2025	Immigration to the United States	Presented to community health center 8 days post-arrival
January 2025	1st hospitalization	See Table 1
February 2025	2nd hospitalization; total IgE 1,664 kU/L	See Table 1
March 2025	3rd hospitalization; allergen panel negative; IgE 1,282 → 1,159 kU/L	See Tables 1, 3
April 2025	4th hospitalization	See Table 1
May 2025	5th hospitalization (age 6); eosinophilia 10%	See Table 1
June 2025	Lost to follow-up	GINA Step 4–5 therapy at discharge; spirometry and biologic evaluation not completed

**Figure 1:**
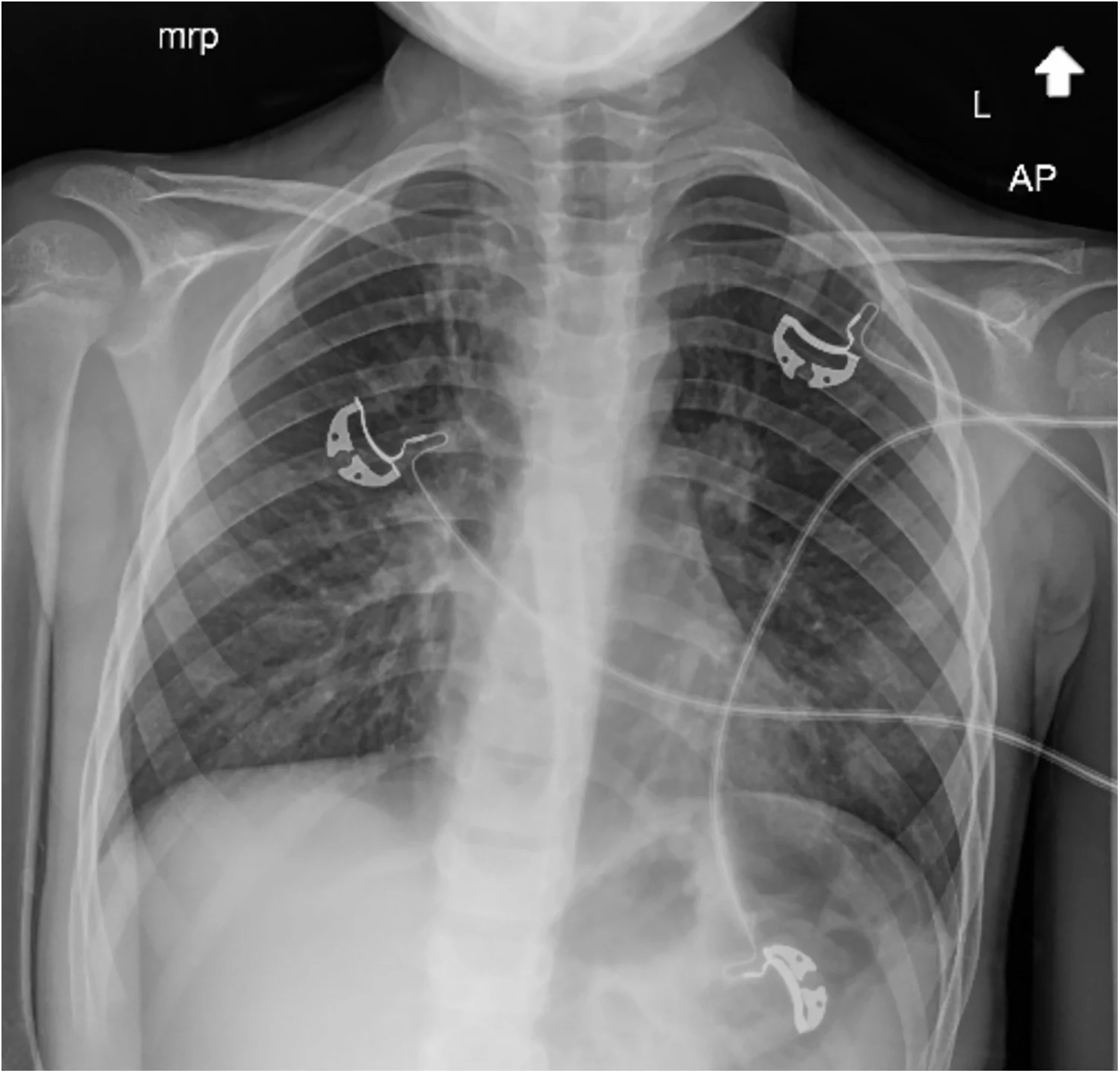
Chest radiograph - anteroposterior chest radiograph demonstrating a small patchy opacity in the medial right upper lobe. No pleural effusion or cardiomegaly is identified.

During her first admission (January 2025), she presented on 10 L supplemental oxygen with diffuse wheezing and a right upper lobe opacity on chest radiograph (Figure 1). Infectious workup was initiated given recent immigration, including QuantiFERON-TB Gold testing and empiric ceftriaxone. Inflammatory markers were normal (CRP 4.2 mg/L, procalcitonin 0.06 ng/mL), and respiratory pathogen panel was negative. She was discharged on as-needed albuterol only.

**Figure 2:**
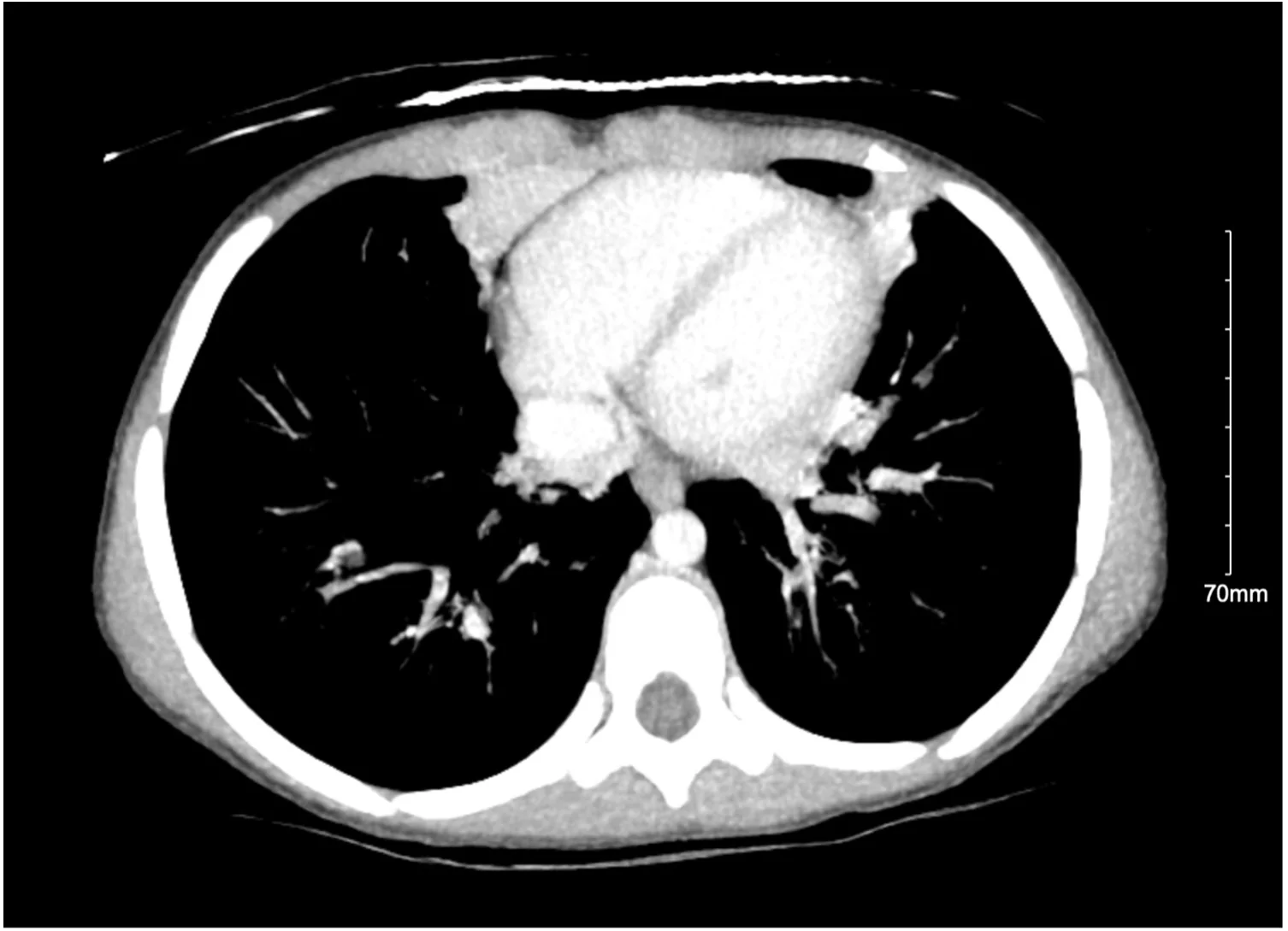
Axial contrast-enhanced CT of the chest at the level of the lower lobes demonstrating diffuse bronchial wall thickening with peribronchial cuffing bilaterally, mosaic attenuation consistent with air trapping, and subcarinal lymphadenopathy. No focal consolidation, pleural effusion, or central bronchiectasis is identified. These findings are consistent with a diffuse small airway inflammatory process in the context of prolonged earthquake rubble dust inhalation.

Her second admission (February 2025) was the most diagnostically revealing. She presented with oxygen saturation of 70%–80% and three episodes of cyanosis the night prior. CT chest demonstrated diffuse bronchial wall thickening, intraluminal debris within several bronchi, ground-glass opacities, and air trapping with reactive mediastinal and hilar lymph nodes (Figure 2). An extensive infectious workup—including QuantiFERON-TB Gold, histoplasma/blastomyces antigens, Coccidioides and Aspergillus panels, hypersensitivity pneumonitis panel, Mycoplasma, and Bordetella—was negative, though rhinovirus/enterovirus was detected on repeat testing. Immunoglobulin levels revealed markedly elevated total IgE at 1,664 kU/L (reference ≤48 kU/L) with mildly elevated IgA (162 mg/dL). Controller therapy was initiated: fluticasone propionate 110 mcg 2 puffs twice daily and montelukast 4 mg daily.

The third admission (March 2025) occurred three weeks later after a missed pulmonology appointment, with oxygen saturation of 77%–80%. Rhinovirus was again detected. Pulmonology consultation raised concern for [allergic bronchopulmonary aspergillosis](/rare-disease/allergic-bronchopulmonary-aspergillosis) (ABPA) given the elevated IgE and earthquake-related dust exposure, and recommended Aspergillus-specific IgG and precipitins, alpha-1 antitrypsin level, and outpatient spirometry. A comprehensive respiratory allergen panel was obtained (Table 3). Tiotropium was planned for initiation at age 6, with biologic therapy under consideration.

**Table 3:** Respiratory allergen panel and Aspergillus-specific testing results.

Test	Result	Interpretation
Aspergillus-specific IgG	Negative	Rules against ABPA
Aspergillus-specific IgE	<0.10 kUA/L	Negative
IgE Alternaria	<0.10 kUA/L	Negative
IgE Cladosporium	<0.10 kUA/L	Negative
IgE Penicillium	<0.10 kUA/L	Negative
IgE Dust Mite (D. farinae)	<0.10 kUA/L	Negative
IgE Dust Mite (D. pteronyssinus)	<0.10 kUA/L	Negative
IgE Cat Dander	<0.10 kUA/L	Negative
IgE Dog Dander	<0.10 kUA/L	Negative
IgE Cockroach	0.17 kUA/L	Class 0/1: Equivocal/Very Low
IgE Mouse Urine	<0.10 kUA/L	Negative
IgE Tree Pollens (Ash, Cedar, Cottonwood, Mulberry, Birch, Elm, Maple, Oak)	All <0.10 kUA/L	Negative
IgE Grass Pollens (Bermuda, Timothy)	All <0.10 kUA/L	Negative
IgE Weed Pollens (Nettle, Russian Thistle, Ragweed, Rough Marsh Elder)	All <0.10 kUA/L	Negative
Total IgE (serial)	1,664 → 1,282 → 1,159 kU/L (Feb → Mar → Mar 2025)	Markedly elevated but trending downward; no allergen-specific sensitization identified

**Figure 3:**
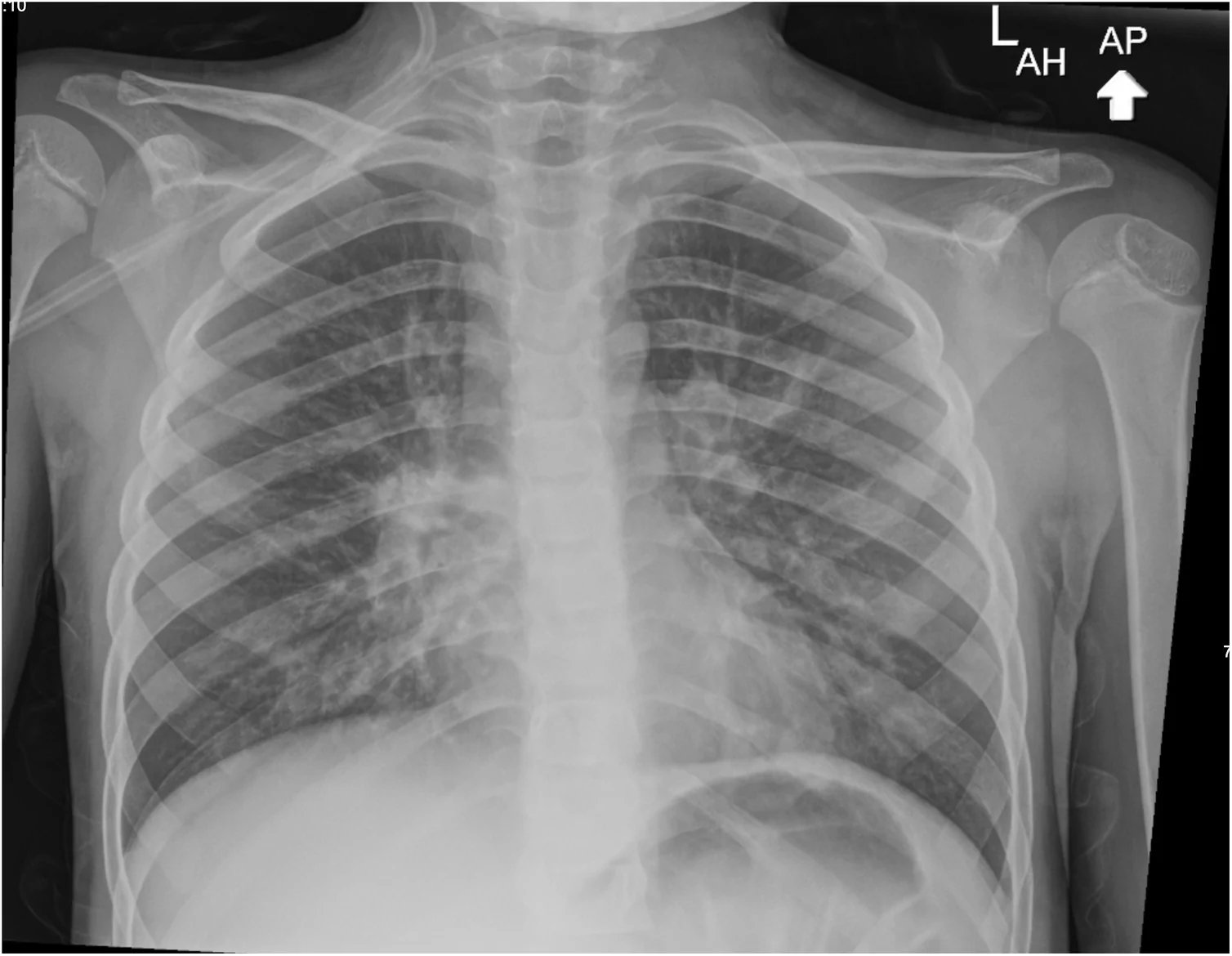
Chest radiograph - anteroposterior chest radiograph demonstrating right middle lobe opacity consistent with atelectasis and bilateral peribronchial thickening.

The fourth admission (April 2025) was directly precipitated by medication nonadherence—the patient had run out of fluticasone two days prior and albuterol the day before. She presented with oxygen saturation of 83%, nasal flaring, tracheal tug, and diminished aeration bilaterally. Chest radiograph showed right middle lobe atelectasis (Figure 3). Social work was engaged to arrange mail-order medications through the patient's insurance to prevent future lapses.

The fifth admission (May 2025), at age 6, occurred one day after running out of tiotropium, with environmental trigger exposure (smoke, marijuana odor, cooking oil scent at school). She required high-flow nasal cannula up to 15 L and could speak only in 2–3 word sentences. Laboratory evaluation now showed peripheral eosinophilia (10%, absolute 0.9 × 10⁹/L), and rhinovirus/enterovirus was again positive. [Cystic fibrosis](/rare-disease/cystic-fibrosis), [primary ciliary dyskinesia](/rare-disease/primary-ciliary-dyskinesia), and alpha-1 antitrypsin testing had all returned negative. Therapy was escalated to mometasone-formoterol (ICS-LABA) 100-5 mcg 2 puffs twice daily, tiotropium was continued, and montelukast was increased to 5 mg daily.

### Current status

At the time of this report, the patient is on mometasone-formoterol 100-5 mcg 2 puffs twice daily, tiotropium 1.25 mcg 2 puffs daily, montelukast 5 mg daily, fluticasone nasal spray daily, and albuterol as needed—corresponding to GINA Step 4–5 therapy. Biologic therapy is under active consideration. She continues to follow with pediatric pulmonology.

## Discussion

This case illustrates the convergence of disaster-related environmental airway injury, complex immunologic activation, and social determinants of health that may have contributed to the development of severe, difficult-to-control asthma in a young child.

### Earthquake dust and airway injury

The patient's three-day entrapment under rubble represents an extreme inhalational exposure. Respirable dust concentrations measured during post-earthquake debris removal in Kahramanmaraş averaged 30.84 mg/m^3^, with scanning electron microscopy identifying asbestos-associated fibrous structures (4). This rubble dust—a complex mixture of pulverized cement, crystalline silica, heavy metals, and fungal spores—may produce oxidative stress, airway hyperresponsiveness, and remodeling. The CT findings of diffuse bronchial wall thickening, intraluminal debris, ground-glass opacities, and air trapping are consistent with a small airway inflammatory process that may be attributable to prolonged high-concentration dust inhalation (5, 6). Epidemiological precedent supports this mechanism: World Trade Center dust exposure was associated with new pediatric asthma diagnoses (aOR 2.3), with children under 5 disproportionately affected (7, 8). A study of 528 children following the Kahramanmaraş earthquakes similarly found demolition dust exposure (aOR 1.9) and temporary housing (aOR 2.1) independently associated with worsening asthma control (10). This patient's exposure intensity—three days of entrapment at age 3 during a critical window of lung development—far exceeds those captured in population-level studies, which may in part explain the severity of her disease. However, the absence of pre-earthquake respiratory and allergic history limits the ability to determine whether this represents new-onset disease or unmasking of pre-existing susceptibility (5, 11).

### Severe asthma versus difficult-to-treat asthma

An important consideration in this case is the distinction between severe therapy-resistant asthma and difficult-to-treat asthma. The GINA guidelines define severe asthma as asthma that is uncontrolled despite adherence with maximal optimized high-dose ICS-LABA treatment and management of contributory factors, or that worsens when high-dose treatment is decreased. Asthma is not classified as severe if it markedly improves when contributory factors such as inhaler technique and adherence are addressed (13). In this patient, two of five hospitalizations were directly precipitated by medication access lapses, and rhinovirus/enterovirus was detected during three admissions. These modifiable factors suggest that the case may represent severe asthma complicated by difficult-to-treat features rather than purely treatment-resistant disease. Full characterization of this patient's asthma severity would require a period of optimized therapy with confirmed adherence, adequate inhaler technique, management of environmental triggers, and objective lung function testing including spirometry with bronchodilator response—none of which could be completed due to loss to follow-up.

### Markedly elevated IgE without allergen-specific sensitization

The patient's markedly elevated total IgE (peak 1,664 kU/L) initially raised concern for [allergic bronchopulmonary aspergillosis] (/rare-disease/allergic-bronchopulmonary-aspergillosis) (ABPA) and severe atopic asthma. However, comprehensive respiratory allergen panel testing did not demonstrate allergen-specific IgE sensitization, including negative testing for Aspergillus species and other common aeroallergens, with only an equivocal cockroach-specific IgE. In addition, Aspergillus-specific IgG was negative. In the absence of Aspergillus sensitization, the diagnosis of ABPA is unlikely, as immunologic evidence of fungal sensitization is a central component of diagnostic criteria (14).

The discordance between markedly elevated total IgE and absent allergen-specific sensitization suggests that this patient's type 2 inflammatory profile may not be driven by classic IgE-mediated allergic mechanisms. Eosinophilia, which was also present in this patient, reflects activation of type 2 inflammatory pathways that may occur in a range of conditions beyond atopic disease, including non-allergic asthma and environmentally triggered airway inflammation. Current asthma frameworks recognize that severe asthma may present with type 2 inflammatory features even in the absence of identifiable allergen sensitization, supporting a broader interpretation of elevated IgE as a marker of immune activation rather than specific atopy (13, 15).

Environmental exposures may further contribute to this pattern. Following large-scale disasters, inhalational exposure to particulate matter and biological debris has been associated with airway inflammation and, in some cases, fungal-related pulmonary disease, even in individuals without prior immunocompromise (5, 14). Although fungal-associated airway disease has been reported after earthquake exposure, this patient's negative Aspergillus-directed testing and lack of characteristic imaging findings argue against a diagnosis within this spectrum (16).

Taken together, the patient's elevated total IgE and eosinophilia are most consistent with a severe asthma phenotype characterized by type 2 inflammation without clear allergen sensitization. These findings highlight the complexity of interpreting immunologic markers in the context of environmental exposure and support a cautious, phenotype-based approach to diagnosis and management rather than reliance on total IgE levels alone.

### Biologic therapy considerations

This patient's persistent uncontrolled asthma despite optimized stepwise therapy, with elevated IgE, peripheral eosinophilia, and recurrent exacerbations, raises consideration for biologic therapy. The patient turned 6 years of age during the hospital course (fifth admission, May 2025), which is clinically relevant because biologic eligibility in pediatric asthma begins at age 6 for most approved agents. Current guidelines emphasize that biologics should be reserved for severe asthma only after confirming adherence, optimizing inhaler technique, and addressing modifiable barriers to care. In this case, recurrent medication lapses and loss to follow-up limited the ability to fully establish treatment-refractory disease under optimal conditions (13, 17).

For children ≥6 years, approved biologic options include anti-IgE (omalizumab), anti-IL-5/IL-5Rα (mepolizumab, benralizumab), and anti-IL-4Rα (dupilumab) (15). Omalizumab is unlikely to be appropriate given the absence of allergen-specific sensitization and IgE exceeding the approved dosing range. The presence of eosinophilia supports consideration of type 2–targeted therapies such as anti-IL-5/IL-5Rα agents or dupilumab, which have demonstrated efficacy in reducing exacerbations in pediatric patients with type 2–high asthma (15).

Optimal biologic selection should be guided by a phenotype-driven approach incorporating biomarkers (eosinophils, FeNO), lung function, and clinical trajectory, as recommended by current guidelines; however, this evaluation was not completed due to loss to follow-up (13, 17).

### Psychological trauma

The patient survived three days of entrapment during which her father died—an event with high potential for post-traumatic stress. A nationwide longitudinal study found PTSD increased asthma risk (HR 2.27), with the youngest age group showing the highest risk (HR 4.01) (18). Among WTC survivors, probable PTSD was associated with 1.65 times greater odds of post-disaster asthma, independent of dust exposure (19). The bidirectional PTSD-asthma relationship involves shared inflammatory pathways, including Th2-skewed immune responses (20). Formal psychological assessment and trauma-informed care should be integrated into this patient's management.

### Social determinants and medication access

Two of five hospitalizations were directly precipitated by running out of controller medications. Potentially avoidable asthma hospitalizations are 63%–113% higher among immigrant children compared to native-born populations (21). The GINA guidelines emphasize that uncontrolled asthma is frequently attributable to modifiable factors—particularly medication availability and adherence—rather than true treatment refractoriness (13). The engagement of social work to arrange mail-order medications was critical, but recurrent lapses underscore the need for systems-level solutions including proactive medication management and community health worker support.

## Limitations

This report has several limitations inherent to the single-case design. Causality between earthquake dust exposure and the subsequent severe asthma phenotype cannot be established; the association is plausible but unproven. Patient-specific exposure measurements (e.g., personal dust monitoring) were not available. Pre-earthquake allergic, atopic, and respiratory history was not obtainable, limiting the ability to distinguish new-onset disease from unmasked pre-existing susceptibility. Family history of atopy or asthma could not be reliably obtained from the surviving parent. The relative contributions of recurrent viral infections (rhinovirus/enterovirus detected on three of five admissions), medication access interruptions (precipitating two of five admissions), ongoing environmental triggers, and psychological trauma to disease severity cannot be precisely quantified. Spirometry—including post-bronchodilator testing—and FeNO measurement were never performed due to loss to follow-up after the fifth admission, precluding objective confirmation of reversible airflow obstruction and full characterization of the patient's asthma phenotype. Parasitic serologies were not obtained, which would be relevant given the elevated total IgE without allergen sensitization in an immigrant child. The patient was lost to follow-up after the fifth admission, preventing assessment of treatment response under optimized therapy, completion of biologic eligibility evaluation, and determination of whether the asthma is truly treatment-resistant versus difficult-to-treat.

## Conclusion

This case illustrates that prolonged entrapment under earthquake rubble during early childhood may contribute to the development of severe asthma characterized by markedly elevated total IgE without allergen-specific sensitization, eosinophilia, and airway inflammatory changes, compounded by recurrent viral triggers, medication access barriers, and likely psychological trauma. The dissociation between extreme total IgE elevation and absent allergen sensitization is a notable finding that may reflect particulate-driven, non-allergen-specific immune activation—a mechanism with experimental support but limited clinical documentation in disaster-exposed children. The relative contribution of each of these factors cannot be determined from a single case. Despite escalation to GINA Step 4–5 therapy, five hospitalizations in five months followed by loss to follow-up underscore both the need for systematic evaluation for biologic therapy eligibility and the profound challenges of maintaining continuity of care in disaster-affected immigrant children, requiring a multidisciplinary approach integrating pulmonology, allergy, psychology, and social work. This case highlights the importance of systematic respiratory screening for pediatric earthquake survivors, analogous to WTC Health Registry programs, and adds to the limited literature on individual-level outcomes following extreme earthquake dust exposure in children.

The CARE Checklist has been completed by the authors for this case report, attached as online supplementary material (22).

## Data Availability

The original contributions presented in the study are included in the article/Supplementary Material, further inquiries can be directed to the corresponding author.
